# PKC, AKT and ERK1/2-Mediated Modulations of PARP1, NF-κB and PEA15 Activities Distinctly Regulate Regional Specific Astroglial Responses Following Status Epilepticus

**DOI:** 10.3389/fnmol.2019.00180

**Published:** 2019-07-24

**Authors:** Ji-Eun Kim, Tae-Cheon Kang

**Affiliations:** Department of Anatomy and Neurobiology, Institute of Epilepsy Research, College of Medicine, Hallym University, Chuncheon, South Korea

**Keywords:** 3CAI, BIM, epilepsy, KN-93, PJ-34, SC79, seizure, U0126

## Abstract

Status epilepticus (SE, a prolonged seizure activity) leads to reactive astrogliosis and astroglial apoptosis in the regional specific manners, independent of hemodynamics. Poly(ADP-ribose) polymerase-1 (PARP1) activity is relevant to these distinct astroglial responses. Since various regulatory signaling molecules beyond PARP1 activity may be involved in the distinct astroglial response to SE, it is noteworthy to explore the roles of protein kinases in PARP1-mediated reactive astrogliosis and astroglial apoptosis following SE, albeit at a lesser extent. In the present study, inhibitions of protein kinase C (PKC), AKT and extracellular signal-related kinases 1/2 (ERK1/2), but not calcium/calmodulin-dependent protein kinase II (CaMKII), attenuated CA1 reactive astrogliosis accompanied by reducing PARP1 activity following SE, respectively. However, inhibition of AKT and ERK1/2 deteriorated SE-induced dentate astroglial loss concomitant with the diminished PARP1 activity. Following SE, PKC- and AKT inhibitors diminished phosphoprotein enriched in astrocytes of 15 kDa (PEA15)-S104 and -S116 phosphorylations in CA1 astrocytes, but not in dentate astrocytes, respectively. Inhibitors of PKC, AKT and ERK1/2 also abrogated SE-induced nuclear factor-κB (NF-κB)-S311 and -S468 phosphorylations in CA1 astrocytes. In contrast, both AKT and ERK1/2 inhibitors enhanced NF-κB-S468 phosphorylation in dentate astrocytes. Furthermore, PARP1 inhibitor aggravated dentate astroglial loss following SE. AKT inhibition deteriorated dentate astroglial loss and led to CA1 astroglial apoptosis following SE, which were ameliorated by AKT activation. These findings suggest that activities of PARP1, PEA15 and NF-κB may be distinctly regulated by PKC, AKT and ERK1/2, which may be involved in regional specific astroglial responses following SE.

## Introduction

Astrocytes are the most abundant glial cells, which participate in a wide variety of complex and essential functions including the maintenance of neuronal excitability (Anderson and Swanson, 2000; Mazzanti et al., 2001) and the homeostasis of extracellular environment as well as metabolism in the brain (Kasischke et al., 2004; Simard and Nedergaard, 2004; Takano et al., 2006). Following various insults, astrocytes show reactive astrogliosis, which inhibits dendritic and axonal remodeling in neuronal circuits, and releases various growth and trophic factors regulating synaptogenesis, neurogenesis, and neuroinflammation after brain injury (Horner and Gage, 2000; Panickar and Norenberg, 2005; Rossi et al., 2007; Shibuya, 2009).

Accompanied by reactive astrogliosis, status epilepticus (SE, a prolonged seizure activity) results in acute and devastating astroglial loss, which is characterized by a pattern of selective vulnerability (Schmidt-Kastner and Ingvar, 1994, 1996). In the hippocampus, SE leads to apoptosis of astrocytes in the molecular layer of the dentate gyrus (referred as dentate astrocytes below; Kang et al., 2006; Kim et al., 2008, 2014, 2017). This SE-induced astroglial apoptosis is relevant to poly(ADP-ribose) polymerase-1 (PARP1) degradation (Kim et al., 2014). Unlike the molecular layer of the dentate gyrus, PARP1 activation is involved in reactive gliosis of astrocyte in the CA1 region (referred as CA1 astrocytes below) where astroglial apoptosis is undetected (Kang et al., 2006; Kim et al., 2014). Basically, PARP1 repairs DNA damage following various injuries. Thus, PARP1 degradation and/or its cleavage lead to apoptosis (Kaufmann et al., 1993; Lazebnik et al., 1994). Since PARP1 utilizes NAD^+^ to form poly(ADP-ribose) polymers (PAR), PARP1 hyperactivation also leads to NAD^+^ depletion and the subsequent failure of bioenergetics that promotes necrotic cell death (Ha and Snyder, 1999; Ying et al., 2002).

Phosphoprotein enriched in astrocytes of 15 kDa (PEA15) is a small phosphoprotein, which is abundantly expressed in astrocytes (Danziger et al., 1995), and protects astrocytes from apoptosis (Estellés et al., 1999; Kitsberg et al., 1999). PEA15 functionality is controlled by phosphorylations: Protein kinase C (PKC) phosphorylates serine (S) 104 site. Calcium/calmodulin-dependent protein kinase II (CaMKII) or AKT preferentially phosphorylate S116 site (Araujo et al., 1993; Estellés et al., 1996; Kubes et al., 1998). Phosphorylated PEA15 accelerates nuclear extracellular signal-related kinases 1/2 (ERK1/2) translocation that activates astroglial proliferation and up-regulation of glial fibrillary acidic protein (GFAP) expression, which are hallmarks of reactive astrogliosis (Liu et al., 2004; Krueger et al., 2005; Meini et al., 2008; Kim and Kang, 2018). Recently, we have reported that the reduced PEA15 expression and its S116 phosphorylation are involved in astroglial apoptosis, while its S104 phosphorylation is up-regulated in reactive CA1 astrocytes following SE (Park and Kang, 2018). Interestingly, protein kinases for PEA15 phosphorylations also reciprocally influence PARP1 activity. For example, PKC inhibits PARP1 (Hegedus et al., 2008), while ERK1/2 activates PARP1 (Kauppinen et al., 2006; Cohen-Armon et al., 2007; Mizuguchi et al., 2011). CaMKII also affects PARP1 enzyme activity (Midorikawa et al., 2006; Goebel, 2009), and PARP1 increases AKT activity (Gerace et al., 2012). Furthermore, both PARP1 and PEA15 regulates nuclear factor-κB (NF-κB) activity (Hassa et al., 2003; Genovese et al., 2005; Stilmann et al., 2009; Wakita et al., 2016), which is is also involved in reactive astrogliosis (Morga et al., 2009). Therefore, it is noteworthy to explore the roles of various kinases in activities of PARP1, PEA15 and NF-κB during reactive astrogliosis or astroglial apoptosis following SE, which remain elusive.

Here, we demonstrate that bisindolylmaleimide (BIM, a PKC inhibitor), 3-chloroacetyl-indole (3CAI, an AKT inhibitor) and U0126 (an ERK1/2 inhibitor), but not KN-93 (a CaMKII inhibitor), attenuated CA1 reactive astrogliosis accompanied by reducing PARP1 activity following SE, respectively. However, 3CAI and U0126 deteriorated SE-induced dentate astroglial loss concomitant with the diminished PARP1 activity. BIM and 3CAI attenuated SE-induced PEA15-S104 and -S116 phosphorylations in CA1 astrocytes, respectively. U0126 and KN-93 did not affect PEA15 phosphorylations in CA1 astrocytes. BIM, 3CAI and U0126 also abrogated SE-induced NF-κB-S311 phosphorylation in CA1 astrocytes. In contrast, 3CAI and U0126 enhanced NF-κB-S468 phosphorylation in dentate astrocytes. Furthermore, PJ-34 (a PARP1 inhibitor) aggravated dentate astroglial loss following SE. 3CAI deteriorated dentate astroglial loss and led to CA1 astroglial apoptosis, which was ameliorated by SC79 (an AKT activator). These findings suggest that PKC, AKT and ERK1/2 may distinctly regulate activities of PARP1, PEA15 and NF-κB in regional specific astroglial apoptosis and reactive astrogliosis following SE.

## Materials and Methods

### Experimental Animals and Chemicals

All animal experimental procedures and protocols were approved by the Institutional Animal Care and Use Committee of the Hallym University (Chuncheon, South Korea). Adult male Sprague–Dawley (SD) rats weighting 250–280 g, were purchased from Daehan Biolink (South Korea). Rats were housed under controlled environmental conditions (23–25 °C, 12 h light/dark cycle) with free access to water and standard laboratory food. All reagents were obtained from Sigma-Aldrich (USA) unless otherwise noted.

### Intracerebroventricular Drug Infusion

Under Isoflurane anesthesia (1%–2% in O_2_ and N_2_O), animals were stereotaxically implanted a brain infusion kit 1 (Alzet, Cupertino, CA, USA) into the lateral ventricle (1 mm posterior; 1.5 mm lateral; −3.5 mm depth; flat skull position with bregma as reference). Thereafter, an infusion kit was connected to an osmotic pump (1007D, Alzet, Cupertino, CA, USA) containing: (1) vehicle; (2) bisindolylmaleimide (BIM, a PKC inhibitor, 25 μM); (3) 3-chloroacetyl-indole (3CAI, an AKT inhibitor, 25 μM); (4) U0126 (an ERK1/2 inhibitor, 25 μM); (5) KN-93 (a CaMKII inhibitor, 25 μM, Santa Cruz, CA, USA); (6) PJ-34 (PARP inhibitor VIII, 3 μM, Merck, Germany); and (7) SC79 (an AKT activator, 25 μM). In a pilot study and our previous studies (Kim et al., 2014; Kim and Kang, 2018; Park and Kang, 2018), each compound treatment did not show behavioral and neurological defects in animals and did not affect seizure threshold in response to pilocarpine. Three days after surgery, animals were used for SE induction.

### SE Induction

SE was induced by a single dose (30 mg/kg) of pilocarpine in rats pretreated (24 h before pilocarpine injection) with 127 mg/kg lithium chloride, as previously described (Kim et al., 2014; Kim and Kang, 2018; Park and Kang, 2018). Before pilocarpine injection, animals were given atropine methylbromide (5 mg/kg i.p.) to block the peripheral effect of pilocarpine. Two hours after SE, animals received diazepam (10 mg/kg, i.p.) to terminate SE. As controls, rats were treated with saline instead of pilocarpine.

### Tissue Processing

Three days after SE, animals were deeply anesthetized with urethane anesthesia (1.5 g/kg, i.p.) and immediately cardiac-perfused with phosphate-buffered saline (PBS, pH 7.4) followed by 4% paraformaldehyde in 0.1 M phosphate buffer (PB, pH 7.4). After perfusion, brains were quickly removed and post-fixed in the 4% paraformaldehyde and cryoprotected by 30% sucrose overnight. Thereafter, the tissues were sectioned with a cryostat at 30 μm and consecutive sections were collected in six-well plates containing PBS.

### TUNEL Staining

According to the manufacturer’s protocol, TUNEL staining was performed using TUNEL apoptosis detection kit (Upstate, Lake Placid, NY, USA). Following the TUNEL reaction, double fluorescent staining was performed (see below).

### Immunofluorescent Study

Sections were incubated in a mixture of appropriate primary antibodies (Table 1) in PBS containing 0.3% Triton X-100 overnight at room temperature. For triple immunofluorescent study, we used M.O.M. kit (Vector, USA, #BMK-2202) according to the manufacturer’s protocol. After washing three times for 10 min with PBS, the sections were also incubated in a mixture of AMCA- (or FITC-) and Cy3-conjugated secondary antisera (or streptavidin, 1:250, Amersham, USA) for 2 h at room temperature. For negative control, the hippocampal tissues were incubated with pre-immune serum instead of primary antibody. All images were captured using an Axio Imager M2 microscope and AxioVision Rel. 4.8 software (Carl Zeiss Korea, Seoul, South Korea).

**Table 1:** Primary antibodies used in the present study.

Antigen	Host	Manufacturer	Dilution used
		(catalog number)	
GFAP	Mouse	Millipore (#MAB3402)	1:500
NF-κB RelA p65-S311	Rabbit	Abcam (ab194926)	1:50
NF-κB RelA p65-S468	Rabbit	Abcam (ab31473)	1:50
PAR	Mouse	Enzo (ALX-804–220)	1:100
PARP1	Rabbit	Abcam (ab32138)	1:100
	Mouse	Trevigen (4338-MC-50)	1:100
PEA15-S104	Rabbit	Antibodies-online	1:200
		(ABIN744683)
PEA15-S116	Rabbit	Antibodies-online	1:200
		(ABIN744698)

### Statistical Analysis

For quantitative analysis of fluorescent intensity, sections (15 sections per each animal) were viewed through a microscope connected *via* CCD camera (Carl Zeiss Korea). Thereafter, fluorescent intensity measurements were represented as the number of a 256-gray scale using AxioVision Rel. 4.8 software (Carl Zeiss Korea). Intensity values were corrected by subtracting the average values of background noise obtained from five image inputs. The optical density was then standardized by setting the threshold levels. In addition, two different investigators performed TUNEL-positive cell counts. All data obtained from the quantitative measurements were tested for the normality and equality of variance. Thereafter, data were analyzed by one-way analysis of variance (ANOVA) coupled with Bonferroni’s *post hoc* test for multiple comparisons. Values are presented as mean ± standard error of the mean (SEM). Differences were considered as significant for *p* < 0.05.

## Results

### Effects of Kinase Inhibitors on Astroglial PARP1 Activity Following SE

**Figure 1:**
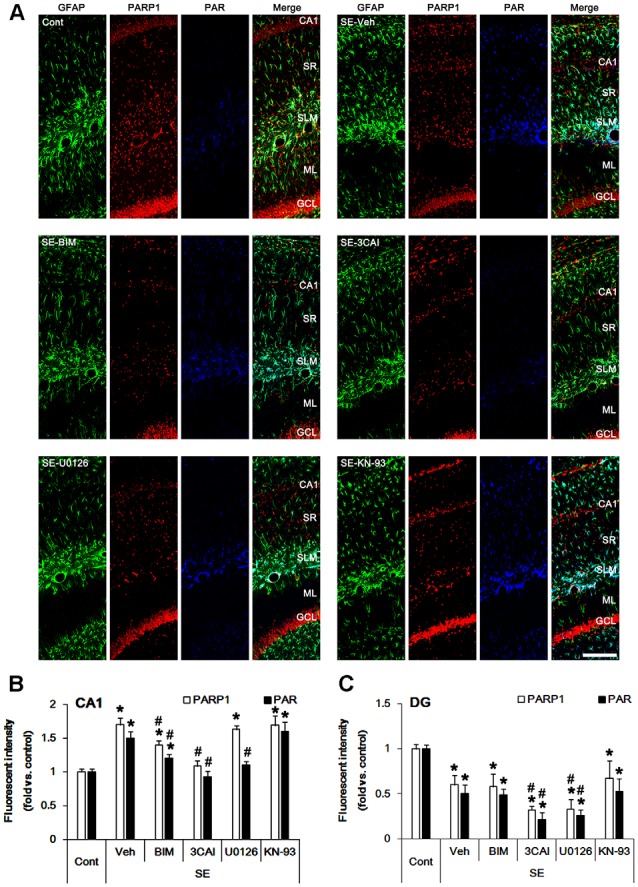
Effect of Status epilepticus (SE) on Poly(ADP-ribose) polymerase-1 (PARP1) expression and PAR level in the hippocampus. SE increases PARP1 expression and PAR level CA1 astrocytes, which are abrogated by BIM and 3CAI. U0126 attenuates the enhanced PAR level without changing PARP1 expression following SE. In contrast, SE diminishes PARP1 expression and PAR level in dentate astrocytes, which are deteriorated by 3CAI and U0126. **(A)** Representative triple immunofluorescent images for glial fibrillary acidic protein (GFAP), PARP1 and PAR following SE. Bar = 100 μm. Abbreviations: CA1, CA1 pyramidal cell layer; SR, stratum radiatum; SLM, stratum lacunosum-moleculare; ML, molecular layer; GCL, dentate granule cell layer.** (B,C)** Quantification of the fluorescent intensities of PARP1 and PAR in CA1 astrocytes **(B)** and dentate astrocytes **(C)**. Error bars indicate standard error of the mean (SEM) (*,^#^p < 0.05 vs. control- and vehicle-treated animals, respectively; *n* = 7, respectively).

First, we validated the characteristics of various kinase inhibitors in regional specific astroglial responses to SE. As compared to control animals, SE led to the increases in PARP1 expression and PAR level in CA1 astrocytes (*p* < 0.05 vs. control; one-way ANOVA, *n* = 7, respectively; Figures 1A,B). As compared to vehicle, BIM (a PKC inhibitor) attenuated the increased PARP1 expression and PAR level in CA1 astrocytes following SE (*p* < 0.05 vs. vehicle, one-way ANOVA, *n* = 7, respectively; Figures 1A,B). 3CAI (an AKT inhibitor) diminished PARP1 expression and PAR level in CA1 astrocytes more than BIM (*p* < 0.05 vs. vehicle and BIM, one-way ANOVA, *n* = 7, respectively; Figures 1A,B). U0126 (an ERK1/2 inhibitor) reduced PAR level without altering PARP1 expression in CA1 astrocytes (*p* < 0.05 vs. vehicle; one-way ANOVA, *n* = 7; Figures 1A,B). However, KN-93 (a CaMKII inhibitor) did not affect the increased PARP1 expression and PAR level in CA1 astrocytes following SE (Figures 1A,B).

Consistent with our previous studies (Kang et al., 2006; Kim et al., 2008, 2017), SE led to massive loss of dentate astrocytes, accompanied by reducing PARP1 expression and PAR level (*p* < 0.05 vs. vehicle, one-way ANOVA, *n* = 7, respectively; Figures 1A,C). U0126 and 3CAI deteriorated dentate astroglial loss concomitant with decreasing PARP1 expression (*p* < 0.05 vs. vehicle, one-way ANOVA, *n* = 7, respectively; Figures 1A,C), while BIM and KN-93 did not affect SE-induced dentate astroglial loss without altering PARP1 expression and PAR level. These findings indicate that PKC, AKT and ERK1/2, but not CaMKII, may regulate PARP1-mediated CA1 reactive astrogliosis and that AKT and ERK1/2 may be also relevant to SE-induced dentate astroglial loss.

### Effects of Kinase Inhibitors on Astroglial PEA15 Phosphorylations Following SE

**Figure 2:**
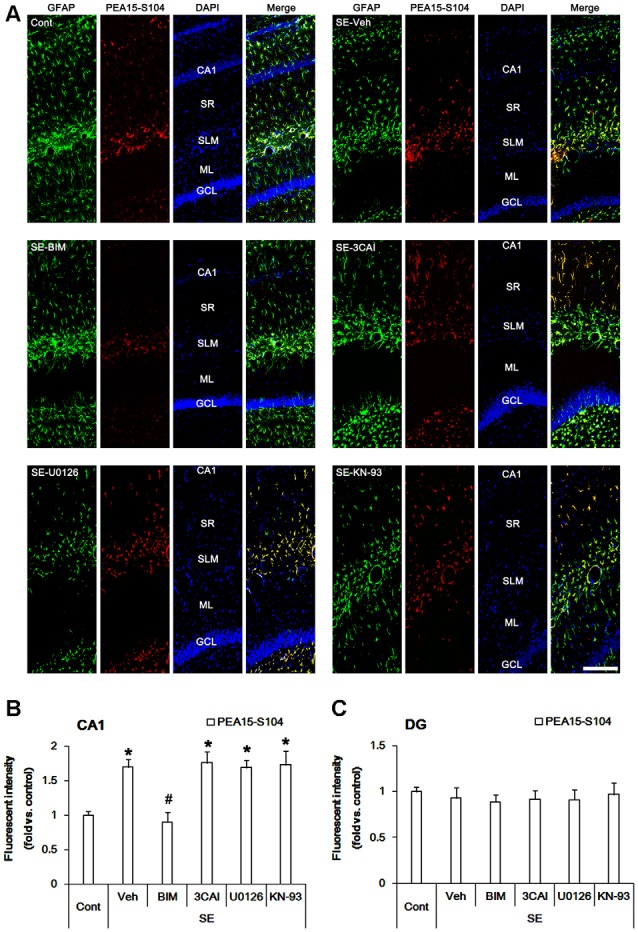
Alterations in PEA15-S104 phosphorylation in the hippocampus following SE. SE enhances PEA15-S104 phosphorylation in CA1 astrocytes, which is reversed by BIM, but not 3CAI, U0126 and KN-93. SE does not influence PEA15-S104 phosphorylation in dentate astrocytes, which is unaffected by BIM, 3CAI, U0126 and KN-93.** (A)** Representative immunofluorescent images for GFAP and PEA15-S104 phosphorylation following SE. Bar = 100 μm. Abbreviations: CA1, CA1 pyramidal cell layer; SR, stratum radiatum; SLM, stratum lacunosum-moleculare; ML, molecular layer; GCL, dentate granule cell layer.** (B,C)** Quantification of the fluorescent intensities of PEA15-S104 phosphorylation in CA1 astrocytes **(B)** and dentate astrocytes **(C)**. Error bars indicate SEM (*,^#^p < 0.05 vs. control- and vehicle-treated animals, respectively; *n* = 7, respectively).

PEA15 expression and its phosphorylations are involved in astroglial response to SE (Park and Kang, 2018). Therefore, we evaluated the effects of kinase inhibitors on PEA15-S104 and -S116 phosphorylations in astrocytes following SE. In control animals, PEA15-S104 immunoreactivity was predominantly observed in CA1 astrocytes (Figure 2A). PEA15-S104 phosphorylation was up-regulated in reactive CA1 astrocytes (*p* < 0.05 vs. control animals, one-way ANOVA, *n* = 7; Figures 2A,B). BIM down-regulated PEA15-S104 phosphorylation level in reactive CA1 astrocytes (*p* < 0.05 vs. vehicle, one-way ANOVA, *n* = 7; Figures 2A,B). 3CAI, U0126 and KN-93 did not affect PEA15-S104 phosphorylation in reactive CA1 astrocytes. As compared to control animals, PEA15-S104 phosphorylation level was unaltered in dentate astrocytes following SE (Figures 2A,C). BIM, 3CAI, U0126 and KN-93 did not influence PEA15-S104 phosphorylation level in dentate astrocytes (Figures 2A,C). These findings indicate that PKC-mediated PEA15-S104 phosphorylation may be relevant to CA1 reactive astrogliosis, while PEA15-S104 phosphorylation may not be involved in degeneration of dentate astrocytes following SE.

**Figure 3:**
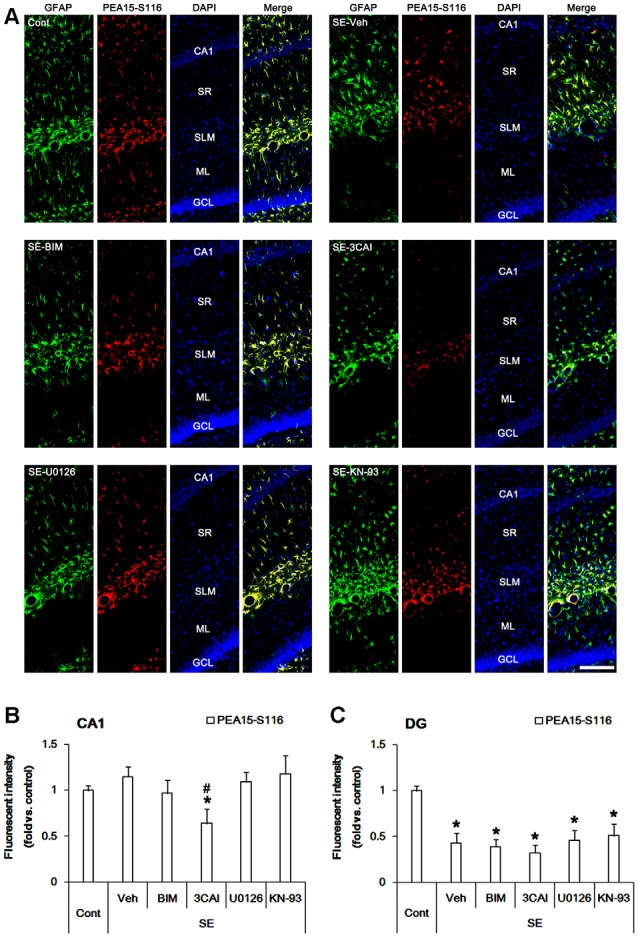
Changes in PEA15-S116 phosphorylation in the hippocampus following SE. SE elevates PEA15-S116 phosphorylation in CA1 astrocytes, which is attenuated by 3CAI, but not BIM, U0126 and KN-93. SE decreases PEA15-S116 phosphorylation in dentate astrocytes, which is unaffected by BIM, 3CAI, U0126 and KN-93.** (A)** Representative immunofluorescent images for GFAP and PEA15-S116 phosphorylation following SE. Bar = 100 μm. Abbreviations: CA1, CA1 pyramidal cell layer; SR, stratum radiatum; SLM, stratum lacunosum-moleculare; ML, molecular layer; GCL, dentate granule cell layer.** (B,C)** Quantification of the fluorescent intensities of PEA15-S116 phosphorylation in CA1 astrocytes **(B)** and dentate astrocytes **(C)**. Error bars indicate SEM (*,^#^p < 0.05 vs. control- and vehicle-treated animals, respectively; *n* = 7, respectively).

In control animals, PEA15-S116 immunoreactivity was detected in CA1 astrocytes and dentate astrocytes. In contrast to S104 phosphorylation, PEA15-S116 phosphorylation was unaltered in CA1 astrocytes following SE (Figures 3A,B). 3CAI reduced PEA15-S116 phosphorylation in reactive CA1 astrocytes (*p* < 0.05 vs. vehicle, one-way ANOVA, *n* = 7; Figures 3A,B), while BIM, U0126 and KN-93 did not influence PEA15-S116 phosphorylation following SE. Unlike CA1 astrocytes, PEA15-S116 phosphorylation was reduced in dentate astrocytes following SE (*p* < 0.05 vs. control animals, one-way ANOVA, *n* = 7; Figures 3A,C). BIM, 3CAI, U0126 and KN-93 did not affect the reduction in PEA15-S116 phosphorylation in dentate astrocytes following SE. Considering CaMKII- and AKT-mediated PEA15-S116 phosphorylation (Araujo et al., 1993; Estellés et al., 1996; Kubes et al., 1998), these findings indicate that AKT, but not CaMKII, may play a role in the maintenance of PEA15-S116 phosphorylation in CA1 reactive astrogliosis following SE.

### Effects of Kinase Inhibitors on Astroglial NF-κB Phosphorylations Following SE

**Figure 4:**
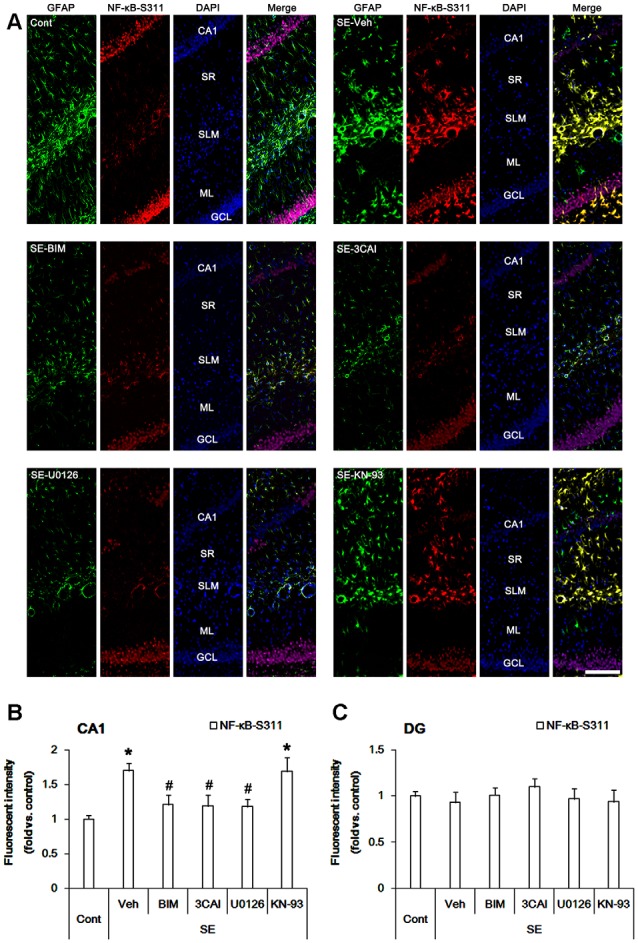
Alterations in NF-κB-S311 phosphorylation in the hippocampus following SE. SE increases NF-κB-S311 phosphorylation in CA1 astrocytes, which is attenuated by BIM, 3CAI, and U0126, but not KN-93. SE does not influence NF-κB-S311 phosphorylation in dentate astrocytes. In addition, NF-κB-S311 phosphorylation is unaffected by BIM, 3CAI, U0126 and KN-93.** (A)** Representative immunofluorescent images for GFAP and PEA15-S116 phosphorylation following SE. Bar = 100 μm. Abbreviations: CA1, CA1 pyramidal cell layer; SR, stratum radiatum; SLM, stratum lacunosum-moleculare; ML, molecular layer; GCL, dentate granule cell layer.** (B,C)** Quantification of the fluorescent intensities of NF-κB-S311 phosphorylation in CA1 astrocytes **(B)** and dentate astrocytes **(C)**. Error bars indicate SEM (*,^#^p < 0.05 vs. control- and vehicle-treated animals, respectively; *n* = 7, respectively).

Since p65 RelA NF-κB (referred as NF-κB below) phosphorylation is also involved in reactive astrogliosis (Morga et al., 2009), we investigated the effects on protein kinase inhibitors on NF-κB-S311 and -S468 phosphorylations that modulate its optimal activity (Viatour et al., 2005). Consistent with our previous studies (Kim et al., 2013), NF-κB-S311 phosphorylation was observed in a few CA1 astrocytes (Figure 4A). Following SE, NF-κB-S311 phosphorylation was increased in CA1 astrocytes (*p* < 0.05 vs. control animals, one-way ANOVA, *n* = 7; Figures 4A,B). BIM, 3CAI and U0126, but not KN-93, attenuated the increased NF-κB-S311 phosphorylation in CA1 astrocytes (*p* < 0.05 vs. vehicle, one-way ANOVA, *n* = 7, respectively; Figures 4A,B). As compared to control animals, NF-κB-S311 phosphorylation was unaltered in dentate astrocytes following SE (Figures 4A,C). BIM, 3CAI, U0126 and KN-93 did not influence NF-κB-S311 phosphorylation level in dentate astrocytes (Figures 4A,C). These findings suggest that PARP1 activation and NF-κB-S311 phosphorylation may play an important role in CA1 reactive astrogliosis.

**Figure 5:**
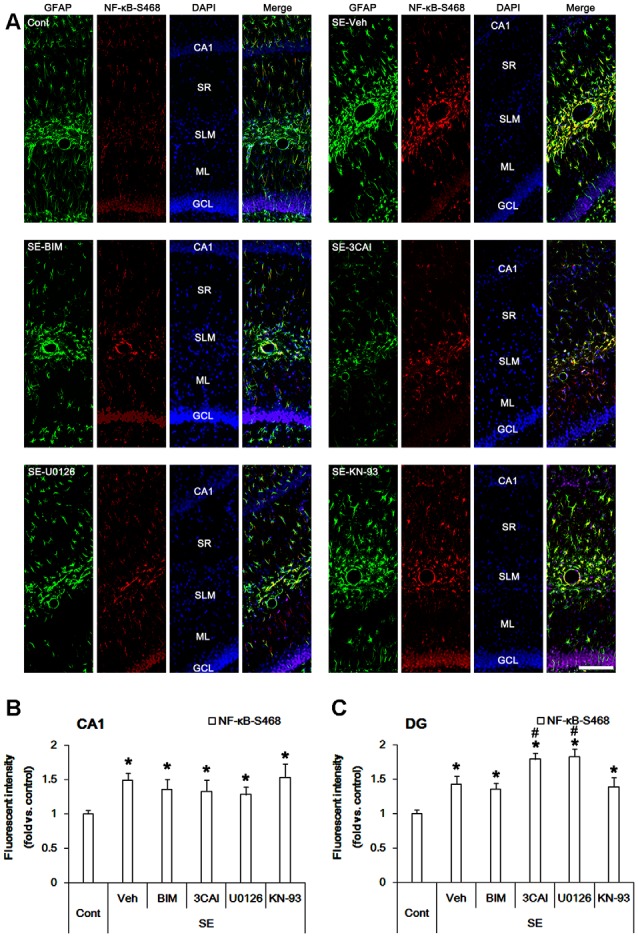
Effect of SE on NF-κB-S468 phosphorylation in the hippocampus. SE increases NF-κB-S468 phosphorylation in CA1 astrocytes, which is unaffected by BIM, 3CAI, U0126 and KN-93. SE also elevates NF-κB-S468 phosphorylation in dentate astrocytes, which is enhanced by 3CAI and U0126.** (A)** Representative immunofluorescent images for GFAP and NF-κB-S468 phosphorylation following SE. Bar = 100 μm. Abbreviations: CA1, CA1 pyramidal cell layer; SR, stratum radiatum; SLM, stratum lacunosum-moleculare; ML, molecular layer; GCL, dentate granule cell layer.** (B,C)** Quantification of the fluorescent intensities of NF-κB-S468 phosphorylation in CA1 astrocytes **(B)** and dentate astrocytes **(C)**. Error bars indicate SEM (*,^#^p < 0.05 vs. control- and vehicle-treated animals, respectively; *n* = 7, respectively).

Similar to NF-κB-S311 phosphorylation, NF-κB-S468 positivity was increased in CA1 astrocytes following SE (*p* < 0.05 vs. vehicle, one-way ANOVA, *n* = 7, respectively; Figures 5A,B). However, BIM, 3CAI, U0126 and KN-93 did not affect NF-κB-S468 phosphorylation in CA1 astrocytes (Figures 5A,B). SE also increased NF-κB-S468 phosphorylation in dentate astrocytes. 3CAI and U0126 enhanced NF-κB-S468 phosphorylation in these cells following SE (*p* < 0.05 vs. vehicle, one-way ANOVA, *n* = 7, respectively; Figures 5A,C), while BIM and KN-93 did not affect it. Therefore, our findings indicate that NF-κB-S468 phosphorylation may be relevant to dentate astroglial loss.

### Effects of Kinase Inhibitors, PARP1 Inhibitor and AKT Activator on Astroglial Viability Following SE

**Figure 6:**
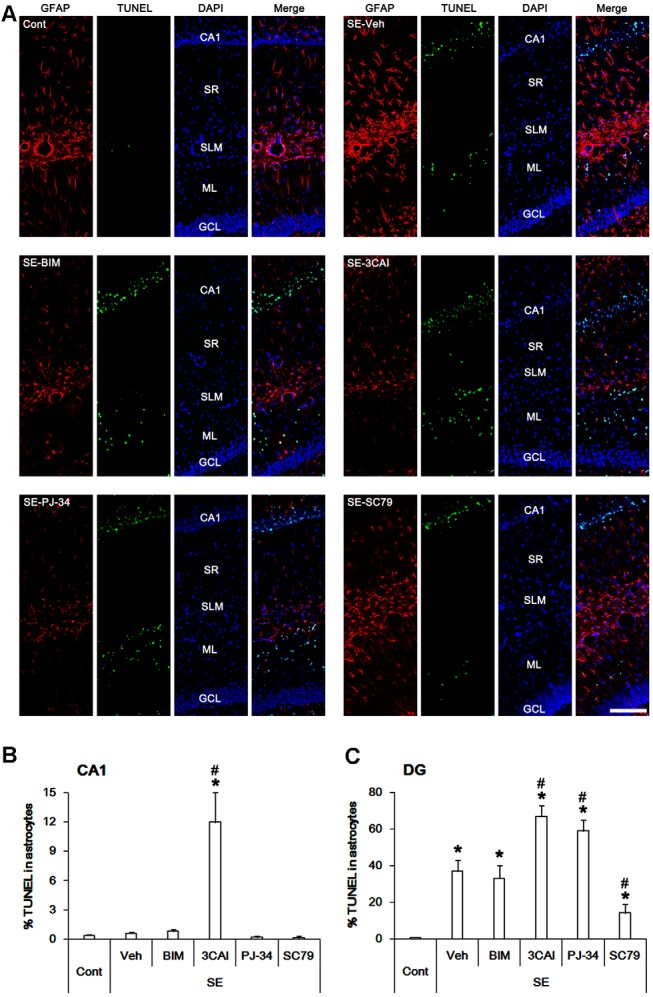
Astroglial apoptosis in the hippocampus following SE. SE does not induce apoptosis in CA1 astrocytes. 3CAI leads to CA1 astroglial apoptosis following SE. However, SE result in apoptotic degeneration in dentate astrocytes, which is deteriorated by 3CAI and PJ-34. SC79 ameliorates dentate astroglial apoptosis following SE. **(A)** Representative immunofluorescent images for GFAP and TUNEL following SE. **(B,C)** Quantification of the percentage of TUNEL positive astrocytes in CA1 astrocytes **(B)** and dentate astrocytes **(C)**. Error bars indicate SEM (*, ^#^p < 0.05 vs. control- and vehicle-treated animals, respectively; *n* = 7, respectively).

Next, we evaluated the effects of kinase inhibitors on astroglial viability in response to SE. TUNEL positive astrocytes were rarely detected in CA1 astrocytes following SE (Figures 6A,B). 3CAI increased the number of TUNEL positive astrocytes in the CA1 region (*p* < 0.05 vs. vehicle, one-way ANOVA, *n* = 7, respectively; Figures 6A,B), while BIM (Figures 6A,B), U0126 and KN-93 (data not shown) did not affect it. To directly elucidate the roles of PARP1 and AKT in astroglial viability, we applied PJ-34 (a PARP1 inhibitor) and SC79 (an AKT activator). Both PJ-34 and SC79 did not influence the number of TUNEL positive astrocytes in the CA1 region (Figures 6A,B). In contrast to CA1 astrocytes, SE significantly increased the number of TUNEL positivity in dentate astrocytes (*p* < 0.05 vs. control animals, one-way ANOVA, *n* = 7, respectively; Figures 6A,C). 3CAI, PJ-34 and U0126 (data not shown) elevated the number of TUNEL positive astrocytes in this region (*p* < 0.05 vs. vehicle, one-way ANOVA, *n* = 7, respectively; Figures 6A,C), while BIM (Figures 6A,C) and KN-93 (data not shown) did not affect it. However, SC79 effectively diminished the number of TUNEL-positive dentate astrocytes following SE (*p* < 0.05 vs. vehicle, one-way ANOVA, *n* = 7, respectively; Figures 6A,C). These findings indicate that PARP1, ERK1/2 and AKT may be involved in astroglial viability in response to SE.

## Discussion

PARP1 plays a different role in reactive astrogliosis and astroglial apoptosis following SE: PARP1 degradation/inhibition evokes astroglial death, but its activation results in reactive astrogliosis (Kim et al., 2014). Although DNA damage is a general cause of PARP1 activation, DNA damage-independent PARP1 activation has been also reported (Kauppinen et al., 2006; Spina-Purrello et al., 2008). Therefore, it is likely that other regulatory signaling molecules for PARP1 activation beyond DNA damage may be involved in the distinct astroglial response to SE, albeit at a lesser extent.

Consistent with our previous study (Kim et al., 2014), the present data show that SE elevated PARP1 expression and PAR level in CA1 reactive astrocytes, which are indicatives of PARP1 activation. Since PARP1 regulates cell proliferation and inflammatory responses *via* various transcription factors such as NF-κB, activator protein-1 and cAMP-response element binding protein (Ha et al., 2002; Hassa et al., 2003), our findings indicate that PARP1 activation may play an important role in CA1 reactive astrogliosis. On the other hand, pilocarpine increases PARP1 expression in CA1 astrocytes of acute brain slice model (Kim et al., 2014). Furthermore, the astroglial proliferation induced by SE is predominantly observed in the CA1 region at 1 week after SE (Kim and Kang, 2018). Taken together, it is likely that PARP1 expression may be up-regulated in naïve CA1 astrocytes rather than newly generated astrocytes.

The present study also reveals that BIM, 3CAI and U0126, but not KN-93, effectively mitigated SE-induced PARP1 activation in CA1 astrocytes. Indeed, PKC increases PARP1 activity in various cell types (Henderson et al., 2017), although PKC-mediated PARP1 phosphorylation protects from DNA damage-induced necrotic cell death (Hegedus et al., 2008). Furthermore, ERK1/2 activates PARP1 is independent of DNA damage (Cohen-Armon et al., 2007), which is required for maximal PARP1 activation (Kauppinen et al., 2006) AKT also regulates PARP1 cleavage-mediated apoptosis (Chiarugi, 2002). With respect to these previous studies, our findings suggest that PKC, ERK1/2 and AKT, but not CaMKII, may modulate PARP1-mediated CA1 reactive astrogliosis. In contrast, down-regulation of PARP1 expression was observed in dentate astrocytes following SE. U0126 and 3CAI aggravated dentate astroglial loss concomitant with decreasing PARP1 expression. In addition, SC79 effectively abrogated apoptosis of dentate astrocytes, while PJ-34 deteriorated it. Thus, our findings indicate that AKT- and ERK1/2-mediated PARP1 activation may play an important role in the viability of dentate astrocytes following SE.

In the present study, BIM reduced PARP1 expression and PAR synthesis level, but U0126 only reduced the PAR level following SE. Although we could not exactly explain these discrepancies in the present study, it is considerable that the different mechanisms/efficacies of BIM and U0126 to inhibit reactive astrogliosis would distinctly affect PARP1 expression and PAR synthesis. This is because PKC regulates reactive astrogliosis and astroglial proliferation (Stanimirovic et al., 1995; Scarisbrick et al., 2012), while ERK1/2 activation is insufficient to induce astroglial proliferation during the process of reactive astrogliosis (Kim and Kang, 2018). Therefore, the differential effects of BIM and U0126 on PARP1 expression and PAR synthesis may result from the distinct underlying mechanisms of BIM and U0126 for inhibiting reactive astrogliosis.

Recently, we have reported that the increased PKC-mediated PEA15-S104 phosphorylation plays an important role in reactive CA1 astrogliosis, while the reduced PEA15-S116 phosphorylation is relevant to apoptosis of dentate astrocytes induced by SE, independent of neuronal damage (Park and Kang, 2018). In the present study, PEA15-S104, but not -S116, phosphorylation was up-regulated in reactive CA1 astrocytes following SE, which was down-regulated by BIM. Since BIM attenuated reactive CA1 astrogliosis induced by SE, it is likely that PEA15-S104 phosphorylation may be involved in reactive astrogliosis. However, 3CAI and U0126 mitigated reactive CA1 astrogliosis concomitant with reducing PARP1 activity, which did not affect PEA15-S104 phosphorylation. Therefore, our findings suggest that PARP1 activation may play a more important role in reactive astrogliosis than PEA15-S104 phosphorylation. The present study also demonstrates that SE diminished PEA15-S116 phosphorylation in dentate astrocytes. BIM, 3CAI, U0126 and KN-93 did not influence PEA15-S116 phosphorylation in dentate astrocytes. However, 3CAI led to CA1 astroglial apoptosis and deteriorated it in dentate astrocytes following SE, which were abrogated by SC79. Considering that PEA15 phosphorylations influence cell viability and PEA15 stability by inhibiting apoptosis and proteasomal degradation (Danziger et al., 1995; Kubes et al., 1998; Trencia et al., 2003; Perfetti et al., 2007), our findings suggest that AKT-mediated PEA15-S116 phosphorylation may play a pro-survival role in astrocytes against apoptosis.

NF-κB phosphorylations transactivate several anti-apoptotic and pro-survival genes (Chiarugi, 2002; Liu et al., 2012). In addition, muscarinic receptor (receptor for pilocarpine) modulates NF-κB translocations and its phosphorylations in astrocytes (Guizzetti et al., 2003), which regulate reactive astrogliosis (Morga et al., 2009). In the present study, SE increased NF-κB-S311 phosphorylation in CA1 astrocytes, which was mitigated by BIM, 3CAI and U0126. These findings indicate that PKC-, AKT- and ERK1/2-mediated signaling pathways may regulate NF-κB-S311 phosphorylation during reactive astrogliosis. The present data also reveal that SE elevated NF-κB-S468 phosphorylation in CA1 astrocytes, which was unaffected by BIM, 3CAI, U0126 and KN-93. Since the phosphorylation of S468 site terminates NF-κB dependent gene expression upon assisting in binding of an E3 ubiquitin ligase complex to NF-κB, which modulates the removal of chromatin-bound NF-κB at promoter sites of a subset of NF-κB genes (Geng et al., 2009; Mao et al., 2009), it is likely that the increased NF-κB-S468 phosphorylation in CA1 astrocytes may be an adaptive response to inhibit reactive astrogliosis. In dentate astrocytes, however, 3CAI and U0126 enhanced the up-regulation of NF-κB-S468 phosphorylation induced by SE. Both AKT and ERK1/2 inhibit glycogen synthase kinase 3β (Cohen and Frame, 2001; Lin et al., 2011), which diminishes NF-κB-S468 phosphorylation (Geng et al., 2009; Mao et al., 2009). In addition, NF-κB inhibition triggers rapid PAPR1 cleavage and subsequent apoptosis (Chiarugi, 2002). Thus, our findings suggest that the NF-κB-S468 phosphorylation may play a pro-apoptotic role in dentate astrocytes.

In the present study, various kinase inhibitors affected activities of PARP1, PEA15 and NF-κB in astrocytes following SE. Since PJ-34 decreases PARP1 and NF-κB expressions independent of DNA damage (Spina-Purrello et al., 2008; Wang et al., 2013), PARP1 expression is positively correlated with NF-κB expression. Indeed, PARP1 directly binds and interacts with NFκB in cellular level (Stanisavljevic et al., 2011), which are regulated by various mechanisms: PARP1 inhibitors reduce NF-κB activity by preventing the degradation of IκB (Genovese et al., 2005; Stilmann et al., 2009). In addition, PARP1-NF-κB interactions synergistically activate transcriptional factors independent of enzymatic activity and DNA-binding ability (Hassa et al., 2003). Therefore, it is likely that PARP1 may interact with NF-κB to increase NF-κB activity, which in turn may modulate NF-κB-dependent gene expression. PEA15 is also involved in NF-κB-dependent transcriptions (Wakita et al., 2016) and regulates PARP1 activity *via* nuclear ERK1/2 translocation (Krueger et al., 2005; Spina-Purrello et al., 2008). With respect to these reports, it is presumable that PARP1-PEA15-NF-κB-mediated framework would differently regulate the regional specific astroglial responses following SE, although it was not directly confirmed in the present study. Further studies are needed to elucidate this hypothesis.

In conclusion, the present study demonstrates that PKC, AKT and ERK1/2 differently regulated activities of PARP1, PEA15 and NF-κB in CA1 astrocytes and dentate astrocytes. Therefore, our findings suggest that the protein kinases may be distinctly involved in regional specific astroglial responses to SE through various signaling molecules, which would hypothesize their networks to regulate astroglial responses to SE.

## Data Availability

All datasets generated for this study are included in the manuscript.

## Ethics Statement

All animal experimental procedures and protocols were approved by the Institutional Animal Care and Use Committee of the Hallym University (Chuncheon, South Korea).

## Author Contributions

J-EK and T-CK designed the project, performed the experiments described in the manuscript, analyzed the data, and wrote the manuscript.

## Conflict of Interest Statement

The authors declare that the research was conducted in the absence of any commercial or financial relationships that could be construed as a potential conflict of interest.
